# Validity and reliability of two alternate versions of the Montreal Cognitive Assessment (Hong Kong version) for screening of Mild Neurocognitive Disorder

**DOI:** 10.1371/journal.pone.0196344

**Published:** 2018-05-23

**Authors:** Adrian Wong, Stanley Yiu, Ziad Nasreddine, Kam-tat Leung, Alexander Lau, Yannie O. Y. Soo, Lawrence Ka-sing Wong, Vincent Mok

**Affiliations:** 1 Department of Medicine and Therapeutics, The Chinese University of Hong Kong, Hong Kong SAR, China; 2 Therese Pei Fong Chow Research Centre for Prevention of Dementia, The Chinese University of Hong Kong, Hong Kong SAR, China; 3 Centre Diagnostique et Recherche sur la Maladie d’Alzheimer, Québec, Canada; 4 Gerald Choa Neuroscience Centre, Lui Che Woo Institute of Innovative Medicine, The Chinese University of Hong Kong, Hong Kong SAR, China; University Of São Paulo, BRAZIL

## Abstract

**Objective:**

Repeated testing using the Montreal Cognitive Assessment (MoCA) increases risks for practice effects which may bias measurements of cognitive change. The objective of this study is to develop two alternate versions of the MoCA (Hong Kong version; HK-MoCA) and to investigate the validity and reliability of the alternate versions in patients with DSM-5 Mild Neurocognitive Disorder (Mild NCD) and cognitively healthy controls.

**Methods:**

Concurrent validity and inter-scale agreement were examined by Pearson correlation of the total scores between the original and alternate versions and the Bland-Altman Method. Criterion validity of the two alternate versions in differentiating patients with Mild NCD was tested using receiver operating characteristic curve (ROC) analysis. One-month test-retest and inter-rater reliability were examined in 20 participants. Internal consistency of the alternate versions was measured by the Cronbach’s α.

**Results:**

30 controls (age 73.4 [4.5] years, 60% female) and 30 patients (age 75.4 [5.5] years, 73% female) with Mild NCD were recruited. Both alternate versions significantly correlated with the original version (*r* = 0.79–0.87, *p*<0.001). Mean differences of 0.17 and -0.40 points were found between the total scores of the alternate with the original versions with a consistent level of agreement observed throughout the range of cognitive abilities. Both alternate versions significantly differentiated patients with Mild NCD from healthy controls (area under ROC 0.922 and 0.724, *p*<0.001) and showed good one-month test-retest reliability (intra-class correlation [ICC] = 0.92 and 0.82) and inter-rater reliability (ICC = 0.99 and 0.87) and high internal consistency (Cronbach α = 0.79 and 0.75).

**Conclusion:**

The two alternate versions of the HK-MoCA are useful for Mild NCD screening.

## Introduction

Mild Neurocognitive Disorder (NCD) is a newly added diagnostic entity recently published in the Diagnostic and Statistical Manual of Mental Disorders, 5^th^ Edition (DSM-5).[1] Mild NCD refers to a syndrome of cognitive decline that is of modest severity with minimal impact on daily functions. While Mild NCD and mild cognitive impairment (MCI) are conceptually similar,[2], Mild NCD encompasses individuals with a milder, possibly earlier stage of cognitive decline compared to MCI. The profile of cognitive domains affected in NCD can be heterogeneous depending on the disease etiology. For example, a predominant learning and memory impairment is expected in patients with NCD due to Alzheimer’s Disease (AD), whereas NCD due to cerebrovascular disease is characterized by executive functions and attentional deficits. Furthermore, the diagnosis of Mild NCD requires a psychometric performance from -1 standard deviation (SD) relative to a matched normative group whereas MCI requires a comparatively more severe impairment from -1.5SD. Screening for mild NCD, therefore, requires cognitive instruments that are sensitive to subtle decline across a comprehensive set of cognitive domains. The Mini-mental State Examination (MMSE) has been reported to be insensitive to detect mild cognitive dysfunction in different neurological disorders and it use is currently fee charging by a commercial company.[3–5] A number of cognitive tests, for example, the Montreal Cognitive Assessment (MoCA),[3] the Oxford Cognitive Screen [6, 7] and the Birmingham Cognitive Screen,[8, 9] have been developed to assess mild cognitive dysfunctions of various etiologies and are currently available in multiple languages.

The MoCA is a 15-minute cognitive test developed for screening of MCI. It includes items to assess a broad range of cognitive domains and abilities including executive functions, visuospatial abilities, language, attention, working memory, abstraction and orientation. The MoCA has been validated in a large number of conditions including stroke, dementia, psychiatric disorders, substance abuse and is increasingly used as the cognitive outcome measure in clinical trials (see http://www.mocatest.org for an updated list of publications) and is one of the most widely used cognitive screening tests around the world.[3] The Hong Kong version of the MoCA (HK-MoCA) is administered in the Cantonese language. The HK-MoCA has been validated and investigated in a variety of clinical conditions including MCI, AD, stroke, cerebral small vessel disease and brain injury [10–15] with age- and education-adjusted normative data available for classification.[16] The HK-MoCA, along with its abbreviated version, the HK-MoCA 5-minute Protocol,[17] are officially listed as the cognitive screening tests for dementia by the Hospital Authority in Hong Kong. To-date, these tests are the most widely used cognitive screen tests in medical and social services in Hong Kong. Cantonese-speaking people constitute an important subgroup of the population in many parts of the world. Over 65 million people in Hong Kong and China speak Cantonese as a first language. Given the rapidly aging population, Cantonese-speaking patients are increasingly encountered in healthcare settings around the world.

With its widespread clinical use, a significant issue inherent in cognitive assessment is the gain of test scores due to an increased familiarity of test stimuli and procedure as a result of repeated exposures to the same test, a phenomenon known as the practice effect.[18] Individuals perform better over repeated testing because they have learned and remembered the test items from previous exposures to the same tests. For example, it has been observed that, on average, individuals with normal cognition gain one point on the MMSE when retested after one year.[19] Practice effects are problematic for longitudinal studies and for monitoring of disease progression or treatment effects because such effects can mask true cognitive changes. Unfortunately, despite the recognition of practice effects, the same version of cognitive test is often used repeatedly in clinical settings and even in large-scale clinical trials. One widely accepted method to circumvent the problem of practice effects is the use of alternate version of the same test. Alternate versions consist of sets of stimuli that are different than that of the original version so that previous exposures to one version of the test have minimal effect upon the performance in other versions. Although alternate versions of the MoCA are available for the English [20, 21] and French versions,[22] there are no published alternate versions for the MoCA in Asian languages.

The objective of this study is to develop two alternate versions of the HK-MoCA and to investigate the concurrent and criterion validity, test-retest and inter-rater reliability as well as internal consistency of the alternate versions in patients with Mild NCD and cognitively normal older adults. We hypothesize that the two alternate versions have adequate psychometric properties for use as a screening instrument for Mild NCD.

## Methods

Sixty participants, including 30 patients with Mild NCD and 30 cognitively normal controls, were recruited as a convenience sample from the Movement and Cognitive Disorders Clinic of the Prince of Wales Hospital and from a pool of 800 functionally independent community older adults recruited in a community study (Risk index for screening subclinical brain lesions in community-dwelling elderly persons in Hong Kong [ref. CUHK471911]).[16] Inclusion criteria for normal controls were normal cognition, as defined by performance within one SD in all cognitive domains (executive functions, learning and memory) measured using the NINDS-CSN VCI 30-minute Neuropsychology protocol (Table 1).[12] Mild NCD was diagnosed according to DSM-5 criteria [1] described in the following: 1) subjective cognitive impairment, defined as ≥1 positive response on the Abbreviated Memory Inventory for Chinese;[23] 2) objective cognitive impairment, defined as performance ≤ -1SD on any of the measure in the NINDS-CSN VCI 30-minute Neuropsychology protocol;[12, 24] and 3) functional independency, defined as a score of ≥2 on Lawton’s Instrumental of Daily Living Scale (IADL).[25] Common inclusion criteria for both groups were 1) aged ≥65 years, 2) adequate sensorimotor and language competency to complete cognitive testing and 3) written informed consent given. Common exclusion criteria were history of stroke or active neurological or psychiatric disorders with known effects upon cognition. This study was approved by the Joint Chinese University of Hong Kong—New Territories East Cluster Clinical Research Ethics committee and written informed consent was obtained from each participant. Study procedures were performed in accordance to the Declaration of Helsinki of 1975 and its later revisions. Permissions for use of the MoCA and the development of the alternate versions as described in the study were obtained from the original author of the MoCA (co-author Dr. Ziad Nasreddine). This study is part of the “*Brain Health Brings Health*” Programme of the Division of Neurology at the Chinese University of Hong Kong.

**Table 1:** Description of the NINDS-CSN VCI 30-minute Neuropsychology protocol.

Cognitive Domain	Test Measure
Executive functions	Symbol Digit Modalities Test (SDMT) total correct[26]
	Animal Verbal Fluency Test total correct
Learning and Memory	Hong Kong List Learning Test (HKLLT)* 10-minute delayed recall/30-minute delayed recall and recognition[27]

*The Hong Kong List Learning Test is a 16-item word list test consisting of 3 learning trials, a 10-minute delayed recall and 30-minute delayed recall and recognition trials.

### Development of alternate versions of HK-MoCA

Two alternate versions of the HK-MoCA, namely HK-MoCA-A1 and HK-MoCA-A2, were developed.[28] An item was replaced in the alternate version if it was judged to be prone to practice effects by expert consensus by a group of healthcare professionals consisting of neurologists, a clinical psychologist and occupational therapists who had extensive experience in using the HK-MoCA in clinical practice. Replaced items were selected for domain-specificity and relevance to the cultural and demographic background of the intended users. A description of the adaptations made in each version is shown in Table 2.

**Table 2:** Modifications in the HK-MoCA alternate versions.

Cognitive domain	*HK-MoCA-A1*	*HK-MoCA-A2*
*Executive/ Activation*	Trails making paradigm: A shape trails paradigm replaces the color trails paradigm. The relative positions of successive connections are changed with the number of steps retained for equal difficulty level.	Trails making paradigm: Relative positions of successive connections are changed.
		Cube is replaced with a rectangular cube
	Clock drawing: time set to 6:10.	Clock drawing: time set to 4:10.
*Naming*	Rhinoceros and camel are replaced by elephant and zebra, respectively.	Lion, rhinoceros and camel are replaced by panda, deer and squirrel, respectively.
*Memory*	5-word learning and memory: All 5 words are replaced by alternative two-syllable words—arm, nylon, shopping mall, rhododendron and orange [color].	5-word learning and memory: All 5 words are replaced by alternative two-syllable words—hair, wool, bank, gladiolus and purple [color].
*Attention*	Digit span test: replaced by alternative digits.	Digit span test: replaced by alternative digits.
	Digit vigilance test: number string replaced by alternative digits with positions of target responses retained.	Digit vigilance test: number string replaced by alternative digits with positions of target responses retained.
	Serial subtraction: “100 minus 7” replaced by “80 minus 7”.	Serial subtraction: “100 minus 7” replaced by “90 minus 7”.
*Language*	Sentence repetition: the two sentences are replaced by sentences “Mom does yoga” and “Teacher teaches writing poems.”	Sentence repetition: The two sentences are replaced by sentences “Mom buys vegetables in wet market” and “Siu Ming [a boy’s name] doing high jumps on the playground.”
	Category fluency: replaced animal category by fruits category.	Category fluency: replaced animal category by vegetables category.
*Abstraction*	Similarities: The word pairs are replaced by “Harmonica—Guitar” and “Calligraphy—Painting.”	Similarities: The word pairs are replaced by “Fork—Chopsticks” and “Pencil—Paper Clips.”

### Data collection

Study procedures were carried out at the designated research clinics at the Prince of Wales Hospital in Hong Kong. Each assessment session lasted within an hour to minimize the possibility of significant fatigue which might confound cognitive test performance. Demographic data including age, sex and years of education were collected. Participants were administrated the NINDS-CSN VCI Neuropsychology 30-minute protocol as part of the screening procedure and were classified into the cognitively healthy control group and Mild NCD group. All participants received the HK-MoCA before the NINDS-CSN VCI Neuropsychology 30-minute protocol. Tests were administered and scored by trained psychometricians according to the published protocols.[26, 27, 29] Each participant received the original HK-MoCA and one of the two alternate versions. Therefore, 30 subjects (regardless of group membership) received HK-MoCA-A1 whereas the remaining 30 received HK-MoCA-A2. The specific alternate version each participant was tested and the order of administration of the original and alternate versions were randomized using computer-generated codes. Administrations of the original and alternate versions spanned one month apart from each other. To assess test-retest reliability of the alternate versions, 20 participants, including 10 controls and 10 patients, were randomly selected and retested with the same alternate version after a one-month period by the same examiner. Inter-rater reliability was examined in 20 randomly selected participants (10 controls and 10 patients) by two different examiners over a one-month period.

### Statistical analysis

Demographic characteristics were compared between patients with Mild NCD and healthy controls using independent sample t test for continuous data and *x*^2^ test or Fisher’s Exact test for dichotomous data. Given the potential confounding effects on cognitive test performance due to the difference in education attainment between the two groups, analysis of co-variance (ANCOVA) with adjustment years of education in addition to age was used to compare the group performance on cognitive test scores. Concurrent validity was measured by Pearson correlation between the total scores between the original (HK-MoCA-O) with the alternate versions. Inter-scale agreement between the total scores of the original and alternate versions was examined using the Bland-Altman Method, which is designed to evaluate the agreement of two continuous measurements.[30] Using this method, the mean difference (i.e. bias) and 95% limits of agreement (LoA) of the total scores between the original and alternate versions were calculated. Smaller inter-scale difference and 95% LoA denote higher inter-scale agreement. Criterion validity of the two alternate versions in differentiating patients with Mild NCD from healthy controls was tested using receiver operating characteristic (ROC) curve analysis.[31] The ROC analysis is a commonly used method for measuring diagnostic accuracy. The Area Under the ROC curve (AUC) represents the chance that when one sample is drawn from a truly normal population and another sample drawn from a truly abnormal population, the score of the normal sample will be higher than that of the abnormal sample (or vice versa, depending on the scoring system). It is an index of diagnostic accuracy and ranges between 0 and 1. AUCs closer to 0 or 1 represent greater accuracy and AUC of 0.5 indicates a complete lack of diagnostic value. Test-retest and inter-rater reliability of the two alternate versions were indexed by the intra-class correlation [ICC]. Internal consistency of items in the alternate versions was measured by the Cronbach’s α. Statistical analysis was performed in R statistical software or IBM SPSS Statistics Macintosh version 24. Figure illustrations were created using Microsoft PowerPoint version 15.30.

Sample size was estimated according to the data from our previous study on HK-MoCA validation conducted in healthy controls and patients with cerebral small vessel disease.[11] A group size of 26 will enable a large effect size [32] measured by Cohen’s *d* of 0.8 with 80% power and α = 0.05 to detect a significant group difference on the HK-MoCA total score. Therefore, a final sample size of 60 was therefore sufficient for this study.

## Results

The demographic characteristics and cognitive performance of the participants are shown in Table 3. All participants were able to communicate in the Cantonese language at a level that is adequate for the test results to be considered valid. No group difference was found in terms of age and sex. The Mild NCD group had significantly fewer years of education when compared to healthy controls.

**Table 3:** Group comparison of demographic characteristics and cognitive performance.

	Healthy Controls	Mild NCD	p
N	30	30	
*Demographic characteristics*			
Age in years	73.4 (4.5)	75.4 (5.5)	0.130
Female n (%)	18 (60%)	22 (73%)	0.273
Education in years	8.1 (5.2)	4.3 (4.3)	0.003
*Cognitive Performance*			
HK-MoCA-O total	23.9 (3.1)	18.9 (3.8)	<0.01
*NINDS-CSN VCI Neuropsychology Protocol*			
SDMT total correct	32.8 (11.7)	20.2 (9.8)	<0.01
Verbal fluency total correct	17.2 (3.5)	12.6 (4.3)	<0.01
HKLLT 10-min delayed recall	7.7 (1.9)	3.6 (1.7)	<0.01
HKLLT 30-min delayed recall	7.3 (1.8)	2.9 (2.1)	<0.01
HKLLT 10-min delayed recognition	13.8 (1.6)	11.6 (2.9)	<0.01

Data shown in mean (standard deviation) unless otherwise specified

Abbreviations: HKLLT—Hong Kong List Learning Test, HK-MoCA-O—Hong Kong Montreal Cognitive Assessment Original version, NINDS-CSN VCI—National Institute of Neurological Disorders and Stroke—Canadian Stroke Network Vascular Cognitive Impairment, SDMT—Symbol Digit Modalities Test

### Concurrent validity

**Fig 1:**
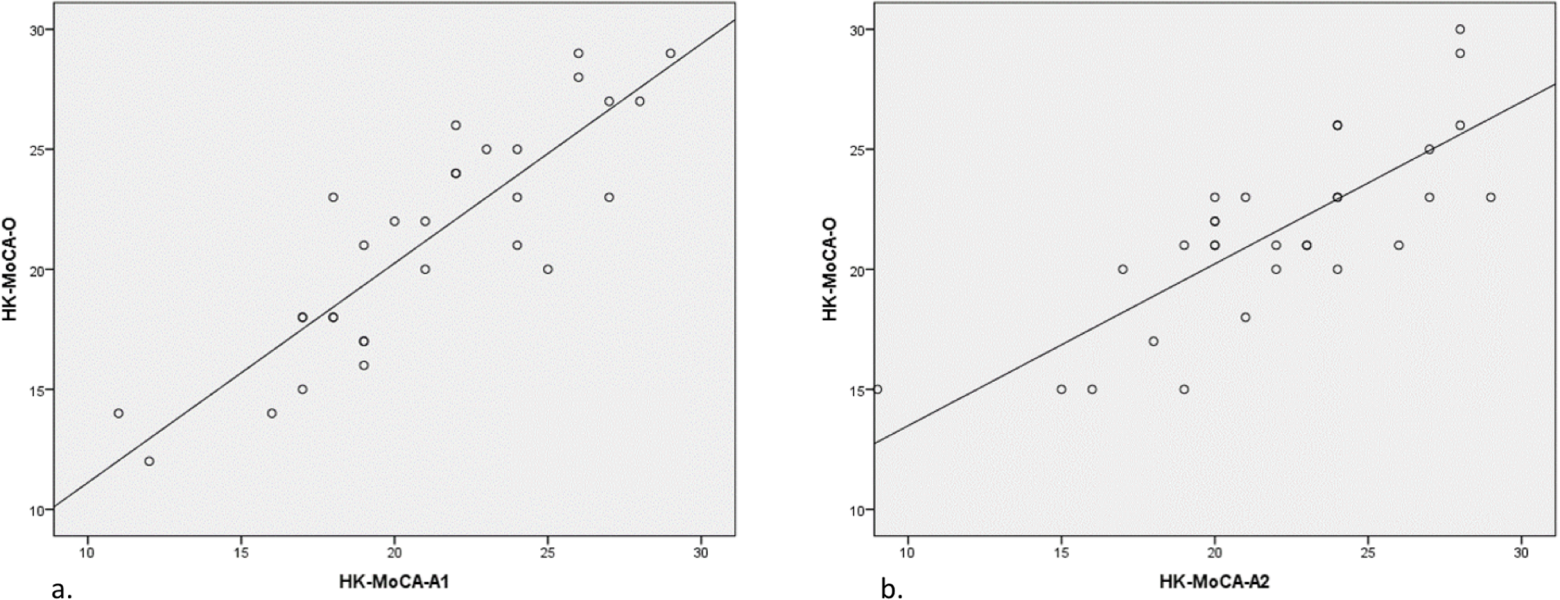
Scatterplots depicting relationships between HK-MoCA-O with HK-MoCA-A1 (panel a) and HK-MoCA-A2 (panel b). HK-MoCA-A1 (*r* = 0.87, *p*<0.001) and HK-MoCA-A2 (*r* = 0.79, *p*<0.001).

Both HK-MoCA-A1 (*r* = 0.87, *p*<0.001) and HK-MoCA-A2 (*r* = 0.79, *p*<0.001) significantly correlated with HK-MoCA-O (Fig 1).

**Fig 2:**
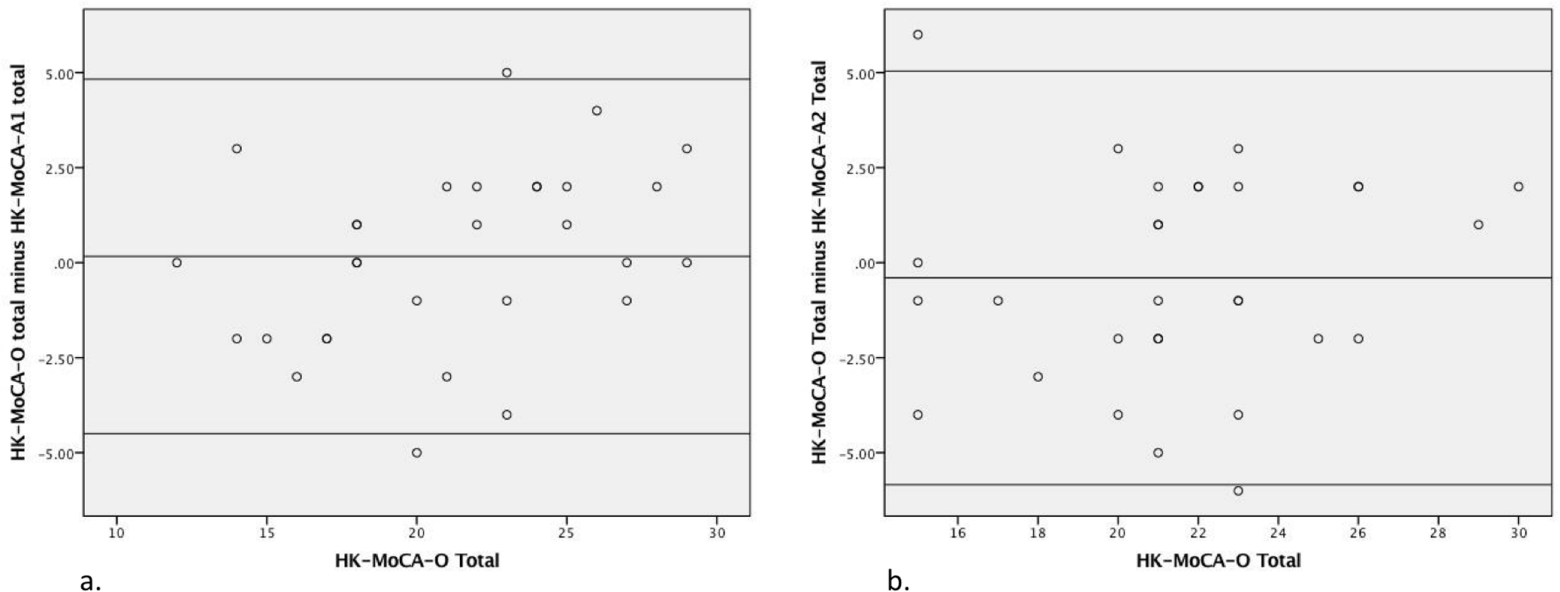
Bland Altman plots showing interscale agreement between HK-MoCA-O with HK-MoCA-A1 (panel 2a) and HK-MoCA-A2 (panel 2b).

Bland Altman analysis revealed a mean difference of 0.17 and -0.40 between the HK-MoCA-O with HK-MoCA-A1 and HK-MoCA-A2, respectively. The 95% LoA was -4.50 to 4.83 for HK-MoCA-A1 and -5.84 to 5.04 for HK-MoCA-A2. Fig 2 shows the Bland Altman plots for the alternate versions. The level of agreement did not appear to differ as a function of cognitive ability measured on the HK-MoCA-O.

### Criterion validity

**Fig 3:**
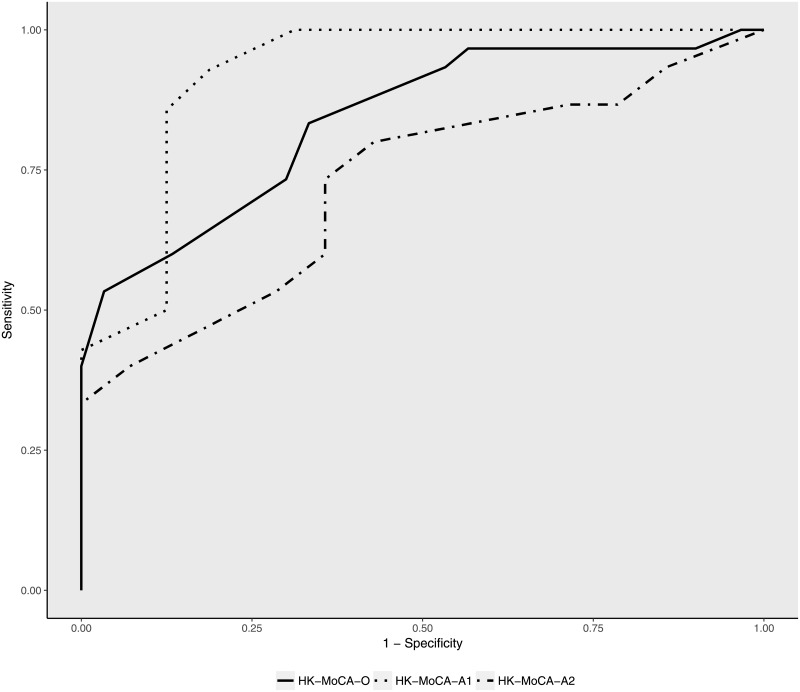
ROC curves for HK-MoCA-O, HK-MoCA-A1 and HK-MoCA-A2 in differentiating patients with Mild NCD from healthy controls. Note that the individual ROC curves are derived from different samples (n = 60 for HK-MoCA-O, n = 30 HK-MoCA-A1 and n = 30 for HK-MoCA-A2 and combined in a single graph as shown here. AUCs are 0.839, *p*<0.001 for MoCA, 0.922, *p*<0.001 for HK-MoCA-A1 and 0.724, *p*<0.05 for HK-MoCA-A2.

ROC analysis revealed AUC of 0.839, *p*<0.001, for HK-MoCA-O in differentiating patients with Mild NCD from healthy controls. Corresponding AUC was 0.922, *p*<0.001 and 0.724, *p*<0.05 for HK-MoCA-A1 and HK-MoCA-A2, respectively. (Fig 3).

### Reliability and internal consistency

ICC for one-month test-retest reliability was 0.92, *p*<0.001 for HK-MoCA-A1 and 0.82, *p*<0.001, for HK-MoCA-A2. ICC for inter-rater reliability was 0.99, *p*<0.001, for HK-MoCA-A1 and 0.87, *p*<0.01, for HK-MoCA-A2. Internal consistency, as measured by the Cronbach’s α, was 0.79 and 0.75 for HK-MoCA-A1 and HK-MoCA-A2, respectively.

## Discussion

In this study, we developed two alternate versions for the HK-MoCA. Both versions demonstrated good concurrent and criteria validity, test-retest and inter-rater reliability as well as internal consistency. In terms of concurrent validity, we showed that both versions significantly correlated with the HK-MoCA-O, with higher correlation observed for the HK-MoCA-A1 (*r* = 0.87, *p*<0.001) than the HK-MoCA-A2 (*r* = 0.79, *p*<0.001). The Bland Altman plot revealed a small mean difference in total score between the original and the alternate version (0.17 and -0.40 point for HK-MoCA-A1 and HK-MoCA-A2, respectively). It also showed a consistent level of agreement throughout the whole range of performance on the HK-MoCA-O, meaning that the difference (bias) between original and each alternate version was similar throughout the entire range of cognitive ability. Note that the mean score difference of the two alternate versions with HK-MoCA-O was 0.17 point for HK-MoCA-A1 and -0.40 point for HK-MoCA-A2. Such differences may be considered minimal for both clinical and research use.

This is also the first study to examine the validity of the MoCA in screening of Mild NCD. We showed that the original and alternate versions are all sensitive to Mild NCD. It is notable that the AUC observed for the HK-MoCA-A1 (0.922) was slightly higher than that of HK-MoCA-O (0.839), indicating that the HK-MoCA-A1 has better discriminating ability than HK-MoCA-A2. Given that the classification of Mild NCD was made partially on the basis of performance on the HK-MoCA-O, the difference in criterion validity between the two alternate versions is possibly explained by the higher correlation with the HK-MoCA-O observed for the HK-MoCA-A1 than the HK-MoCA-A2. Therefore, despite that both alternative versions are valid and reliable, preference is given to HK-MoCA-A1 in view of the better psychometric properties of this version over HK-MoCA-A2.

There are study limitations. First, the sample size was relatively small. However, this sample size was estimated *a priori* based on previous data of the HK-MoCA and it is shown to be sufficient for the analyses performed. Second, participants was recruited based on convenience sampling and therefore the possibility of selection bias could not be excluded. Third, objective psychometric performance for the diagnosis of Mild NCD was measured using the 30-minute protocol of the NINDS-CSN VCI Harmonization battery. While this protocol covers executive functions and memory which are impaired early in the common cognitive disorders such as Alzheimer’s and vascular diseases, participants with isolated impairments in other cognitive domains such as visuospatial functions and attention may have been misclassified as normal. Fourth, in this study we did not determine sensitivity and specificity for a specific cut-off score for the alternate versions. We have previously derived age- and education- adjusted normative values for the HK-MoCA-O from a large cohort of functionally independent old adults free of significant MRI abnormalities.[16] In view of the high correlation observed between the alternate and original versions, it is reasonable to apply the same set of normative data to the alternate versions for classification of performance rather than relying on a single cut-off score. Fifth, Mild NCD was diagnosed clinically. Without information on neuroimaging and biomarkers such as those obtained in *in vivo* amyloid imaging or cerebrospinal fluid analysis, the etiology of the Mild NCD patients was not determined. Finally, it is common for elderly people in Hong Kong to speak one or more Chinese dialects other than Cantonese which could have influenced how the administration instructions and items of the tests were processed. Although we did not include a detailed assessment of the dialects the participants spoke, all of them were able to understand and speak Cantonese at a level well enough so that the test results could be considered valid.

In conclusion, the two alternate versions of the HK-MoCA are useful for screening of Mild NCD. The availability of the alternate versions of the HK-MoCA is expected to improve accuracy in measuring cognitive changes for monitoring disease progression and rehabilitation and treatment efficacy in clinical practice and research studies. Future investigations may focus on using digital technology to capture subtle human behaviors such as voice, response latency and multidimensional movement data to improve sensitivity and accuracy as well as to reduce administration time of cognitive screening.

## Supporting information

S1 Dataset Minimal data set. The minimal data set contains all the data pertinent to the analysis and results reported in this manuscript.
(SAV)
